# Supplementary material for: Patterns of Intron Gain and Loss in Fungi
Source: PLoS Biol. 2004 Nov 30;2(12):e422. doi: 10.1371/journal.pbio.0020422 (PMC532390; doi:10.1371/journal.pbio.0020422)
Supplement: Table S1 — Also available at http://genes.mit.edu/NielsenEtAl/. (4.3 MB ZIP). [file pbio.0020422.st001.zip › NielsenEtAl/html/750.html]

AN3916.1.NCU06005.1.MG01281.1.FG03247.1


```
 CLUSTAL W (1.82) Multiple Sequence Alignments - Introns Inserted


Sequence 1: AN3916.1	608 aa
Sequence 2: FG03247.1	516 aa
Sequence 3: NCU06005.1	657 aa
Sequence 4: MG01281.1	577 aa
Alignment Length: 742 aa
Number Identitical Residues: 266 aa
Alignment Score (without introns) 12635


MG01281.1 	------------------------MAASVSTCGIATP----PSRPS-~--------RVPS
NCU06005.1	MSPSMWTMRWSHQVINEHHRHHLKSCSTISRVNNALKSIIQRTRPGD1TNTDTPHEALPS
FG03247.1 	-----------------------------------------------~------------
AN3916.1  	-----------------------------------------------~------------
          	                                                            

MG01281.1 	T-LGSKPSRMHDPDIIINALKDVVLPAVALSAQHVEN--------------------AQV
NCU06005.1	TPLEPVPTRTTSVTMKLLNLNDGVEAGVMCVEPTLTNDGMDARNLAKAHKGPIVTHLEHL
FG03247.1 	------------------------------------------------------------
AN3916.1  	------------------------------------------------------------
          	                                                            

MG01281.1 	P-----SPDPEPEDMYIGSIDQGTTSSRFIIFNRAGEPVEAHQIEFENRYPNSG~WHEHD
NCU06005.1	PRGIHETPEERKNHWFVGSVDQGTTSSRFLIFNGEGDPVASHQIEFENLYPKSG~WHEHD
FG03247.1 	----MPLLNGKANTAFIGAIDQGTTSSRFLIFDPSGDVVATHQIEFKQYYPHPG2WHEHD
AN3916.1  	---------MDPAKIFVGSIDQGTTSTRFLIFNREGDPVASHQVEFKQIYPNPG2WHEHD
          	               ::*::******:**:**:  *: * :**:**:: **:.* *****

MG01281.1 	PMELLSSVEECIEEAMIKFTATRPG-AKVHSVGITNQRETTVVWDTETGEPLYNAVVWPD
NCU06005.1	PMTLLNSVEECIEGAMRKFQRLGFSKNDIRSIGITNQRETTLVWDHETGEPLYNAVVWSD
FG03247.1 	PEELISSVEQCIDGAVETFESQGHSREQIKAVGITNQRETTVVWDKTTGKALHNAIVWTD
AN3916.1  	PLELVSSVETCIEEAVKQFEINGYDQQSIKAVGITNQRETTVVWDHETGEPLYNAIVWTD
          	*  *:.*** **: *:  *     .  .::::*********:***  **:.*:**:**.*

MG01281.1 	TRTSPIVHELRQKPGAEDLLDLCGLPLSTYPSSVKLMWLLRNVDTVKAAYDAGRLAFGTV
NCU06005.1	TRTKSLVRDLKDKDGADAIKDLCGLPLSTYPSSVKLLWLIENVEAVKQAYEEGRLAFGTV
FG03247.1 	TRSKDLVRRLKRRLGSSELIGRCGIPLSTYPSVSKLLWLLDNIPEVKDAYERGVLAFGTV
AN3916.1  	TRSQAVVDELKKKPGASQLQQICGLPLSTYSSSSKLLWMLTNVPKVKEAYEKGTLAFGTV
          	**:. :*  *: : *:. :   **:*****.*  **:*:: *:  ** **: * ******

MG01281.1 	DSWLIYRLTG-----KSVHVTDGSNASRTMFMNLRSMSYDEKLLSFFEIDATKIRLPEIV
NCU06005.1	DTWLIYKLNGGAQAESPVHVTDSTNASRTMFMNIRTLQYDDKMLGFFGIDRTKVHLPKIV
FG03247.1 	DTWLTYKLNGAT--DRDVYVSDPSNASRTMFMNLETLKYDEDILDWFRIDDSKITLPRIV
AN3916.1  	DAWLVYRLNGGS--AANVFVSDCTNASRTMFMNLETLGYDDFLLDFFGIKG-RVHLPKIV
          	*:** *:*.*.:     *.*:* :*********:.:: **: :*.:* *.  :: **.**

MG01281.1 	PSAHPTAFGKFASGPLKDVRIAGCLGDQSSALVGQRAFASGQAKNTYGTGCFLLYNVGPE
NCU06005.1	PSSDPECFGRIASGALSGVPIAGCLGDQSSALVGQCGFNPGQAKNTYGTGCFLLYNVGTE
FG03247.1 	RSSDNKAYGSLAGTSLKGVKITGCLGDQSAALVGQKAFTPGLAKNTYGTGCFLLYNVGTK
AN3916.1  	PSSDVTAYGALSNGRLAGVPIMGCLGDQSSALVGQKGFSPGMAKNTYGTGCFLLYNVGDK
          	 *:.  .:* ::.  * .* * *******:***** .* .* **************** :

MG01281.1 	PVISKAGLLATVAYDFGEELGGRAYALEGSIAVAGSGVKFLSNNLGFAPSSGEITDLAGS
NCU06005.1	PVISQYGLLSTVAYDFGQGR-APVYALEGSIAVAGAGVTFLQNNLNFIENAKQINDLAES
FG03247.1 	PVISSHGLLTTVAFDFGEGN--TMYALEGSIAVAGSSVKFLVDNFGFIESSAKLSALAET
AN3916.1  	PVISKHGLLATIAYNFD-GR--PVYALEGSIAVGGSGIKFLQNNLEFFRESKEVNDLALT
          	****. ***:*:*::*.       *********.*:.:.** :*: *  .: ::. ** :

MG01281.1 	VDDNGGVVFVTAFSGLFAPYWIDDAKGTLF1GLTQHTQKGHIARATLEATCYQTKAILDA
NCU06005.1	VPDNGGVVFVTAFSGLFAPYWIDDAKGTLF1GITQHTQKGHIARATLEATCFQTKAILDA
FG03247.1 	VEDNGGCTFVTAFSGLFAPYWIDDARGTIF~GITSYTQRGHIARATLEATCFQTKAILDS
AN3916.1  	VEDNGGCVFVTAFSGLYAPYWIDDAKGTIF1GITQYTQKGHIARATLEATCFQTKAILNA
          	* **** .********:********:**:* *:*.:**:************:******::

MG01281.1 	MERDSGRKLERLAVDGGMSNSDLCMQ~TQADVGNIPVDRPEMRETTALGAAIAAGLATGV
NCU06005.1	MEKDSGCKLKLLAVDGGLSNSDLTMQ~TQADISGIPVDRPAMCETTALGAAIAAGLATGV
FG03247.1 	MEKDSGHALTELAVDGGMCNSDLIMQ0TQSDLIGIPVNRPGMRETTALGAAIAAGFAVGI
AN3916.1  	MEKDSGHALFELAVDGGMSNSDLAMQ0IQADLISIPVYRPKMRETTALGAAIAAGLAVGL
          	**:***  *  ******:.**** **  *:*: .*** ** * ************:*.*:

MG01281.1 	WKNISEVLEVGGRNRSQFLPSPDRAKAQRMFKKWEQAVEMSRGWLREGEE----------
NCU06005.1	WSDTTELQEVNRTGRKVFEPKISRKCAEKRFKKWNQAVQMSRGWLQDNDNEDDECDEEVK
FG03247.1 	WKSFDDLKEVNTQGRTIFKPQIAEEEATKRFGRWTKAVEMSKGWANE-------------
AN3916.1  	WRNFAELRDINRAGGTVFRPQTSQDISREQFSLWEKAVSMCRGWVGDKSHATEHRDTEGK
          	* .  :: ::.  . . * *.  .  : . *  * :**.*.:**  : .   .  . . .

MG01281.1 	------------------------------------------------------------
NCU06005.1	KA----------------------------------------------------------
FG03247.1 	------------------------------------------------------------
AN3916.1  	PTQKVDLSNGHKGVVDAASATAPSNIVSPLLSAKMNGDLVLSKGTPWINISGDLEDADDQ
          	 :.. . :.. ..  .::::::.:.  :.  ::. ...   :..:.  . :.. ..:...

MG01281.1 	--------------------------
NCU06005.1	--------------------------
FG03247.1 	--------------------------
AN3916.1  	DLFLELRKLEILQRLKRLGKVKVTYF
          	.   .  . .  .  .  .. . :
```
